# LotuS: an efficient and user-friendly OTU processing pipeline

**DOI:** 10.1186/2049-2618-2-30

**Published:** 2014-09-30

**Authors:** Falk Hildebrand, Raul Tadeo, Anita Yvonne Voigt, Peer Bork, Jeroen Raes

**Affiliations:** 1 Department of Structural Biology, Vlaams Instituut voor Biotechnologie (VIB), Pleinlaan 2, Brussels 1050, Belgium; 2 Department of Bioscience Engineering, Vrije Universiteit Brussel, Pleinlaan 2, Brussels 1050, Belgium; 3 Structural & Computational Biology Unit, European Molecular Biology Laboratory (EMBL), Meyerhofstrasse 1, Heidelberg 69117, Germany; 4 Molecular Medicine Partnership Unit (MMPU), University of Heidelberg and European Molecular Biology Laboratory, Heidelberg, Germany; 5 Max Delbrück Centre for Molecular Medicine, Robert-Rössle-Str. 10, Berlin 13125, Germany; 6 Department of Microbiology and Immunology, REGA institute, KU Leuven, Herestraat 49, Leuven 3000, Belgium; 7 VIB Center for the Biology of Disease, Herestraat 49, Leuven 3000, Belgium

**Keywords:** OTU, 16S rDNA gene, Pipeline, Metagenomics, Demultiplexing

## Abstract

**Background:**

16S ribosomal DNA (rDNA) amplicon sequencing is frequently used to analyse the structure of bacterial communities from oceans to the human microbiota. However, computational power is still a major bottleneck in the analysis of continuously enlarging metagenomic data sets. Analysis is further complicated by the technical complexity of current bioinformatics tools.

**Results:**

Here we present the less operational taxonomic units scripts (LotuS), a fast and user-friendly open-source tool to calculate denoised, chimera-checked, operational taxonomic units (OTUs). These are the basis to generate taxonomic abundance tables and phylogenetic trees from multiplexed, next-generation sequencing data (454, illumina MiSeq and HiSeq). LotuS is outstanding in its execution speed, as it can process 16S rDNA data up to two orders of magnitude faster than other existing pipelines. This is partly due to an included stand-alone fast simultaneous demultiplexer and quality filter C++ program, simple demultiplexer (sdm), which comes packaged with LotuS. Additionally, we sequenced two MiSeq runs with the intent to validate future pipelines by sequencing 40 technical replicates; these are made available in this work.

**Conclusion:**

We show that LotuS analyses microbial 16S data with comparable or even better results than existing pipelines, requiring a fraction of the execution time and providing state-of-the-art denoising and phylogenetic reconstruction. LotuS is available through the following URL: http://psbweb05.psb.ugent.be/lotus.

## Background

Next generation sequencing platforms are reducing the cost of collecting metagenomic data from large environmental and clinical microbial ecosystems. With 16S rDNA amplicon sequencing becoming a mainstream approach in these research areas, there is a need to optimize computer resources to handle this data.

Although online services exist to process 16S rDNA data such as the Ribosomal Database Project (RDP) pipeline [1] or the PyroTagger pipeline [2], a single HiSeq run can yield up to 6 × 10^9^ sequences^1^, which challenges uploading capabilities. Large initiatives, like the Human Microbiome Project (HMP), have used the 16S pipelines Quantitative Insights Into Microbial Ecology (QIIME) [3] and mothur [4], applications that can be installed and run locally. mothur follows the philosophy of incorporating all tools in one software package, while QIIME (partly) relies on 3rd party software. Both pipelines present a complete package with tools to interpret the community composition, but more work-intensive components like denoising and sequence clustering are designed for cluster environments.

**Figure 1:**
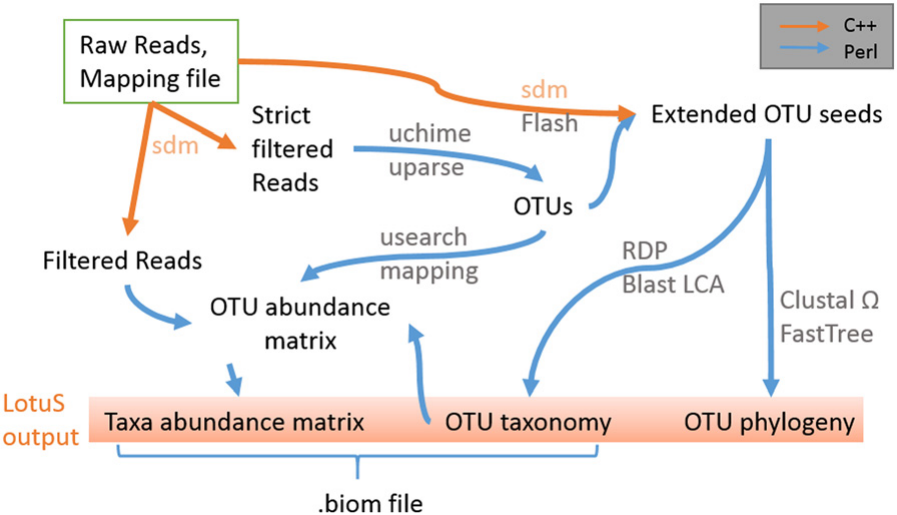
**Overview of the LotuS workflow.** Raw reads are demultiplexed and quality filtered; from these, OTUs are clustered. Mid- and high-quality reads are mapped to OTUs; the taxonomy and phylogenetic relatedness are calculated on the extended OTU seeds.

We developed less OTU scripts (LotuS) as an open-source pipeline in Perl and C++, which clusters operational taxonomic units (OTUs), generates taxonomic-level abundance matrices and a phylogenetic tree of the OTUs directly from non-demultiplexed sequencing files (Figure 1). Currently, 454, HiSeq and MiSeq technologies are supported. Two popular proprietary programs, UPARSE [5] and RDP classifier [6], were incorporated to cluster OTUs, remove sequencing noise and chimeric sequences and classify OTUs taxonomically. Alternatively, OTU seed sequences can be classified by BLAST lowest common ancestor (LCA) comparison to either greengenes [7] or SILVA 16S rDNA databases [8]. LotuS installation does not require root access or changes in system paths and is automatically performed with a script that can additionally also update LotuS to newer versions. This script downloads, installs and configures all proprietary software and databases as well as configuring LotuS to these, with the exception of UPARSE, which requires user registration. Tutorials about LotuS usage and subsequent R numerical analysis are provided on the LotuS webpage (http://psbweb05.psb.ugent.be/lotus/).

## Implementation

### Sequence filtering

Sequences are filtered with a novel C++ program bundled with the pipeline, sdm, that has evolved over the course of this project from a simple demultiplexer to a general purpose sequence file formatting, quality filtering/adjustment and OTU sequence (“Seed”) picking tool, optimized for speed and a high recovery rate of sequences. Further benefits of this program include the simultaneous sorting of input sequences into “high-” and “mid-” quality sequences, dependent on the overall quality and length. These two sequence bins will be important in the further LotuS workflow: the high quality sequences are used in the sensitive OTU clustering process, reducing the number of spurious OTU’s caused by sequencing errors and not biological diversity. Both, high- and mid-quality sequences are used to count the occurrence of OTU’s in single samples, with the intent that mid-quality sequences, which are mapping to an established OTU, will not confound overall diversity measures but add to the count of given biological entities present in a sample.

Default sdm options for 454 and MiSeq sequences are provided with LotuS; these can be modified to filter input sequences after average quality, accumulated error over the sequence, quality in a freely definable window and remove 5′ low-quality bases filtered for these criteria. Furthermore, sequences are filtered for min/max length, ambiguous nucleotides, max barcode and primer errors, polynucleotide runs, and trimmed for adapter sequences, if present. sdm/LotuS accepts fasta + quality, fastq and gzipped versions of these as input. Furthermore, sdm can be used on non-demultiplexed sequences to do a quality filtering of sequences, e.g. prior to assembly [9,10].

### OTU clustering

Filtered sequences are clustered into OTUs with UPARSE. UPARSE is implemented as described by [5], with the exception that the “-cluster_OTUs” command is executed with the additional parameters -uparse_maxhot 62 -uparse_maxdrop 12 that increase the number of potential best hits that are explicitly aligned. This makes the overall clustering slightly slower, but in our experience, it results into more consistent OTUs on MiSeq datasets in case studies, thereby reducing the total number of OTUs. Also, we noticed that default UPARSE can propose a minor fraction of OTUs that are overlapping with other OTUs (mapped within the desired OTU sequence similarity, e.g. 97%, to existing OTUs). OTU abundance is estimated by mapping mid- and high-quality filtered input sequences onto the newly created OTUs.

### Seed extension

The OTU sequence, here termed OTU seed sequence, should fulfil the following criteria to represent the OTU for sequence matching, taxonomic annotation and tree reconstruction: it should be as long as possible, be close to the median of all amplicons clustered to the OTU (representing the centre of the OTU) and contain the least amount of sequencing errors. One common practice is to use a consensus sequence of all amplicons clustered to an OTU, also used by UPARSE. However, a consensus sequence could be the average of two or more strains that constitute the OTU and the UPARSE denoising algorithm is critically dependent on sequences pruned to a reduced length (typically 250bp for 454 sequencing). To resolve this, sdm searches within all high quality input sequences for a sequence matching the above criteria, in a process we call “Seed extension”. In brief, all input sequences are aligned with the consensus OTU sequence using usearch [11]. From these, sdm selects iteratively the hit being closest to the OTU median, having the highest overall mean accumulated error and the longest overall sequence length, with low-quality 5′ nucleotides being removed. In the case of paired MiSeq or HiSeq sequences, the highest-quality pair is selected and these are then merged using flash [12].

### Taxonomic annotation of OTUs

By default, the OTU taxonomy is derived from RDP naïve bayes classifier annotations [6]. The minimum acceptance confidence for RDP is by default set to 0.8 but can be modified via “-rdp_thr”. Alternatively, extended OTU seed sequences are aligned against a reference 16S rDNA database with BLAST + [13]; currently, we support greengenes [7] and SILVA [14] 16S rDNA databases, but this is easily extendable to other ribosomal databases. A lowest common ancestor algorithm is used to assign a taxonomy modified to only consider hits within 1.5% sequence identity to the overall best hit to the current OTU seed, with a limit of 200 total hits. This threshold is chosen to allow for sequencing errors to still include relevant hits, while limiting the space of possible hits within a close enough range to the current best hit; it was validated on 200 bp simulated reads (see Additional file 1) and is a good trade-off between precision and specificity of taxonomic assignments (Additional file 2). At each taxonomic level, the taxonomic consistency of each hit is evaluated. By default, a taxonomic assignment is accepted if >90% of references are the same taxa, modifiable with the LotuS parameter “-LCA_frac”. If a reference has no taxonomic information for a given level (and taxonomic assignments were consistent thus far), it is discarded. In an extreme example, this can lead to species-level taxonomic assignments even if only one reference is assigned to species level but all other references have no taxonomic information.

Furthermore, the sequence similarity of the best hit is used to delimit the taxonomic depth of an assignment. Even if the best hit has a known species name, if the identity of the OTU to the reference is 96%, the taxonomy will only be used up to genus level, as a best reference at a 96% threshold could indicate that this OTU is not represented by a reference species in the database. The default parameters are to limit species at 97%, genus at 95%, family at 93%, class at 91%, order at 88% and phylum at 78% sequence similarity, though users can change these parameters as the established 16S rRNA gene taxonomy does strictly speaking not follow consistent cutoffs for different taxonomic levels [15,16].

### Multiple alignment and phylogenetic reconstruction

The calculation of a phylogenetic tree of the extended OTU seed sequences is an optional step. The phylogenetic tree can be used to fulfil requirements for calculating diversity indices such as Faith’s phylogenetic diversity [17] or between sample UniFrac distances [18]. For this step, the sequences are aligned with Clustal Ω [19] with default parameters for nucleotide alignments. From the aligned sequences, a phylogenetic tree is reconstructed using the gamma model of sequence evolution (options “-nt -gamma -no2nd -fastest -spr 4”) in FastTree2 [20], as recommended by its author (http://www.microbesonline.org/fasttree/) and is saved in Phylip format.

### LotuS Output

LotuS saves the output in the specified output folder in a structure that can be directly integrated into specialized analytical packages of statistical software packages. OTU abundance matrix, the phylogeny for each OTU, a phylogenetic tree and a .biom formatted OTU matrix are stored in this folder. Three subfolders contain a) the run logs and processing reports, b) copies of configuration files and c) higher level taxa abundance matrices.

## Results and discussion

We used a simulation of 1000 greengenes [7] 16S sequences, truncated and randomly mutated (see Additional file 1), to validate the LCA algorithm and research the influence of read length on taxonomic classification. This showed that longer reads are assigned with a higher confidence in RDP (Additional file 3a) and the fraction of 16S reads that remains unclassified using our LCA algorithm significantly decreases with longer read length, when using a reference database from which the 1,000 simulated reads were removed (Additional file 3b). With reads ≥250 bp, our simulation converged to 100% precision and specificity (Additional file 4a,b). To simulate a rare situation where no close relatives are present in the reference database, we used the LCA algorithm only on database hits that had <97% identity to the target read. Here the fraction of taxonomic assignments is in general lower and no species-level assignments were made, as expected given default parameters (Additional file 3c). Precision and specificity of assignments were lowered, and here, the longer read lengths were especially important (Additional file 4c,d).

Read quality is decreasing with increasing read length in 454 and illumina sequencing [5], and UPARSE improves OTU clustering by using only the high-quality 5′ DNA. The here proposed OTU seed extension is important for taxonomic classification and tree building, especially for badly characterized species. It takes advantage of the improved, fast OTU clustering, while using long, high-quality reads for taxonomic annotations and multiple sequence alignments.

We tested the validity and performance of LotuS on cecal gut samples from five different mice strains [21]. It consists of two 454 GS FLX runs and a total of 393,070 reads, the expected read length is 400-500 nucleotides. We used five methods (described in Additional file 1) to calculate OTU abundance matrices: LotuS using RDP taxonomy (LR), LotuS using BLAST taxonomy (LB), QIIME *de novo* OTU creation (QDN), QIIME with reference-based OTUs (QRE) and mothur (MOT). LotuS was run in 454 mode:

lotus.pl -i [path to fasta/qual] -o [output dir] -m [mapping file] -s [sdm option file]

**Figure 2:**
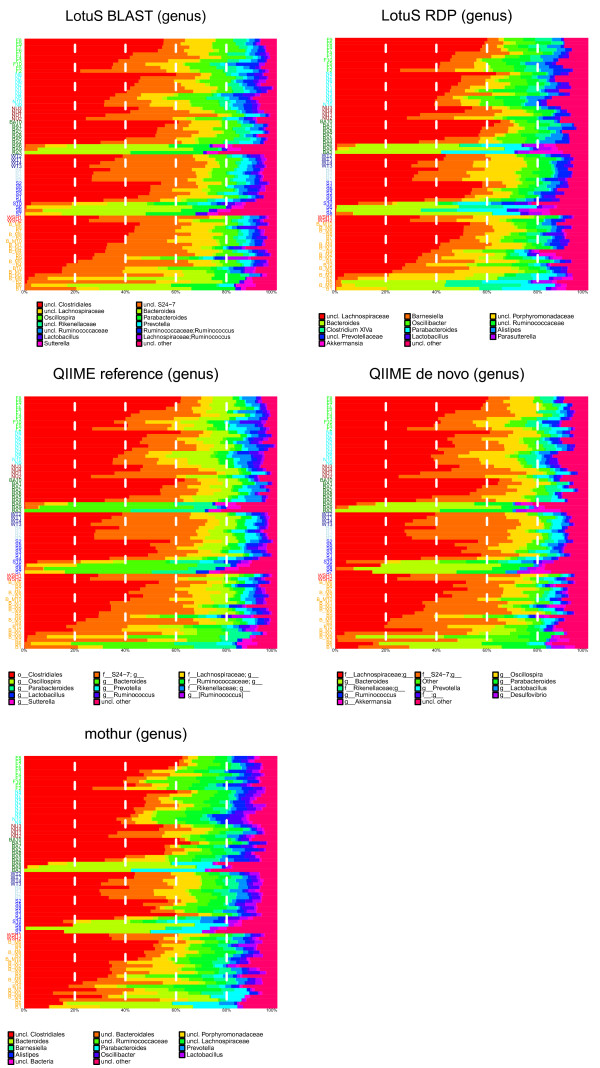
**Genus level compositional comparison.** Comparison of mice caecal composition in example datasets between the five methodologies used. *Y*-axis are single samples; the percentage of reads that is assigned to specific genera is displayed on the *x*-axis.

74 samples were demultiplexed, each sample containing 4699 ± 742 reads. 43,555 reads were rejected due to the low quality filtering criteria. Median OTU seed length was 513 nucleotides for LotuS with a median quality of 37.2. For QDN, OTU seed sequences are slightly longer (527 bp), because the longest available sequence with no quality clippings and filtering is used. In mothur, this was 248 bp, because only high-quality and informative sequence parts were retained, similar to the 250 bp cutoff used by LotuS to cluster OTUs. OTU and taxon-read abundance matrices from these three pipelines were processed in the R statistical computer language. In general, the sample composition is very consistent between the five methods (Figure 2), when taking into consideration differences in taxonomy due to the database/assignment algorithm, that were different in all three pipelines. To measure how well the sample relationships are maintained if different pipeline versions are used, the correlations between Bray-Curtis (BC) and weighted UniFrac (wUF) inter-sample distances were compared in an approach similar to the Mantel test [22] (Additional file 5: Table S1 and Additional file 6: Table S2). The average correlation was 0.993 ± 0.005, 0.988 ± 0.001 and 0.958 ± 0.02, on OTU BC, wUF and genus BC distances, respectively.. Both LotuS-derived wUF were closer to those of mothur than QRE or QDN; they were also closer to QRE than QDN, where the guide tree is based on the greengenes phylogenetic tree, indicating that our *de novo* constructed guide tree is similar to the greengenes one. OTU diversity varied strongly between methods [5]: QDN and QRE clustered most OTUs (6,148 and 1,467), followed by mothur (913) and LotuS (369) (Table 1). When rarefying samples to 2,000 reads, the average OTU number across all samples was similar between LotuS and mothur, with 119 and 118 OTUs/sample. QIIME-derived data had higher sample diversity, with 243 and 201 rarefied OTUs in QDN and QRE, respectively. The total number of reads in the abundance matrices differs only slightly between pipelines (345,801 ± 9,622).

**Table 1:** Richness comparisons between pipelines

	**LB**	**LR**	**QDN**	**QRE**	**MOT**
s_obs	119.2243	118.6378	243.1324	201.8838	119.0432
Chao1	142.314	141.8862	473.0673	273.9279	151.7102
Evenness	0.778795	0.778613	0.752087	0.807766	0.763974
Shannon	3.698957	3.690516	4.128862	4.253904	3.627188

Average diversity and richness estimates for the five methods to derive an OTU matrix, rarefied to 2,000 reads per samples. *LB* LotuS BLAST, *LR* LotuS RDP, *QDN* QIIME *de novo* OTU creation, *QRE* QIIME reference OTU picking, *MOT* mothur.

The number of unassigned taxa at a given taxonomic level is usually lowest in QRE and highest in QDN. QRE does not use a LCA algorithm to exclude cross hits and this is obvious at genus level where only 54.7% of taxa are undefined, which is lower than LB (65.9% of genera unassigned, Additional file 2). QRE is by default excluding sequences that do not match a reference with <97% and the unassigned taxa are thus from the greengenes taxonomic assignment. From the known genera, 55% are shared between all methods, and this applies for 71% of families (Additional file 7). Execution times are the most outstanding difference between tested pipelines (Table 2). The same dataset was processed with all pipelines using the same 2.7 GHz Intel Core i7 MacBook (1 core). Default LotuS was 25 to 200 times faster than the other pipelines and demultiplexing and quality filtering with sdm was 4–6 times faster than these processes in other pipelines. However, when matching reads to a reference database, LotuS is limited by the speed of BLAST+.

**Table 2:** Computational efficiency

**Dataset**	**Time (s)**	**Lotus_RDP**	**Lotus_BLAST**	**QIIME DN**	**QIIME Ref**	**mothur**
2 × 454	Demultiplexing/quality filtering	37	37	160	160	235
2 × 454	Full run	177	7,317	4,325	17,081	39,660
2 × MiSeq	Demultiplexing/quality filtering	820	820	3,495	3,495	*
2 × MiSeq	Full run	8,856	23,761	69,696	56,916	*

Execution times for the five pipelines in seconds performed on the same computer. The output of “full runs” is OTU abundance, higher taxonomic level abundance and a phylogenetic tree of OTUs. The table is further separated into the execution times on our 454 mice faeces test set (2 × 454) and the two human faeces MiSeq test sets (2 × MiSeq). Asterisks denote that mothur was excluded, due to an unknown error (see Additional file 1).

One of the main problems in testing the validity of metagenomic processing is that the real taxonomic composition of a metagenomic sample is unknown or only available for artificial datasets [23], and thus, the absolute error due to bioinformatics processing is hard to estimate. Here we sequenced 40 technical replicates in two separate MiSeq runs to circumvent this problem, as we can determine a relative error between replicates that could be attributed to a pipeline. In this dataset, LotuS recovered 28,789,221 reads from the two MiSeq runs, 19% or 10% more reads than QRE or QDN, respectively. The average sample richness at 15,000 rarefied sequences was highest in QDN (1,333 ± 393.5) followed by LotuS (720.5 ± 186) and closed-reference OTU picking QRE (469.6 ± 146). This could be related to QRE being too stringent as not all OTUs might be present in reference database and QDN being too lenient. Comparing the reproducibility of technical replicates between the two MiSeq runs, we used Canberra, Bray-Curtis and Jensen-Shannon distances between technical replicates. The mean of these was measured for each pipeline variant and on OTU, genus and family level. Overall, no consistent trend was detected for one pipeline recovering more reproducible compositions that any other (Table 3). Comparing the reproducibility of richness, LotuS OTU matrices (identical between LR and LB) had an average richness difference of 5.7 ± 5.9%, lower compared to QRE (7.5 ± 6%, *p* = 0.0065 compared to LotuS) and QDN (6.4 ± 6.3%, *p* = 0.17 compared to LotuS), thus richness was more stable with LotuS among technical replicates. Runtime was again fastest in LotuS (Table 2), though differences in execution time were not as extreme as observed for the 454 dataset with LR being ~8 times faster than Qiime.

**Table 3:** Compositional similarity of technical replicates

OTU	LR	LB	QR	QD
Bray-Curtis	0.117267	0.1158	0.106167	0.153467
Canberra	0.037446	0.037591	0.038509	0.036918
Jensen Shannon	0.001487	0.001572	0.001602	0.001651
Genus	LR	LB	QR	QD
Bray-Curtis	0.0496	0.048133	0.044133	0.045933
Canberra	0.055303	0.055628	0.05864	0.05339
Jensen Shannon	0.002053	0.002067	0.002236	0.001977
Family	LR	LB	QR	QD
Bray-Curtis	0.046867	0.043333	0.042833	0.044933
Canberra	0.060573	0.057641	0.065774	0.063415
Jensen Shannon	0.002129	0.002124	0.002432	0.002502

The technical replicates between two MiSeq runs were compositionally compared, using Bray-Curtis, Canberra and Jensen Shannon distance metric. The table shows the average distance of 38 pairs of replicates, for each pipeline execution mode. Less distance means more similar replicate samples.

## Conclusions

The novel LotuS pipeline is able to handle small to very large 16S datasets on a personal computer and effortlessly integrate multiple sequencing runs. Computational efficiency is very high due to a selection of state-of-the-art proprietary software like UPARSE for denoising and sequence clustering and sdm for demultiplexing and sequence filtering. Comparison to other pipelines suggests a high similarity in higher taxonomic composition to existing tools, but on OTU level, the *de-novo*-called OTU shows an increased richness compared to closed-reference OTU calling and less richness than non-denoised *de novo* OTU calling, as expected. This pipeline has the advantage of state-of-the-art, flowgram-independent denoising and long, high-quality OTU sequences from the OTU seed extension step used for phylogenetic tree construction and taxonomic annotation.

## Endnote

^1^http://www.illumina.com/systems/hiseq_comparison.ilmn

## Availability and requirements

**Project name:** LotuS, sdm.

**Project home page:**http://psbweb05.psb.ugent.be/lotus

**Operating system(s):** Linux, Mac

**Programming language:** Perl, C++

**Other requirements:** proprietary software, downloaded by autoinstaller, UPARSE

**License:** GNU GPL

**Any restrictions to use by non-academics:** licence needed.

## Abbreviations

*sdm*: simple demultiplexer; *LotuS*: less OTU scripts; *OTU*: operational taxonomic unit; *rDNA*: ribosomal DNA.

## Competing interests

The authors declare that they have no competing interests.

## Authors’ contributions

FH designed and implemented the pipeline. Pipeline validity was tested by FH and RT. Samples for the MiSeq runs were collected, extracted and sequenced by AYV. FH, RT, AYV, PB and JR wrote the manuscript. All authors read and approved the final manuscript.

## Supplementary Material

Additional file 1 **Supplementary methods.** Supplementary methods including the following sections: sample collection and 16S rRNA sequencing, 16S read simulation, comparison of pipelines and commands used to run mothur and QIIME on sample datasets. Click here for file

Additional file 2 **Dependency of precision and specificity of BLAST-based LCA from best hit subset.** The reference database sequences included for LCA evaluation are dependent on % identity to best hit; by default all reference sequences that have an identity ≤1.5% of the best found hit are included (red line). Specificity and precision are dependent on this parameter and the default 1.5% is a trade-off between a high precision and a high specificity. Click here for file

Additional file 3 **Classification performance is dependent on 16S read length.** 16S reads (1,000) of different length were simulated from the greengenes database (Additional file 1). a) RDP average classification confidence on six taxonomic levels that is increasing constantly with increasing read length. b) Similarly, the fraction of simulated reads that were not assigned to a taxon, using our LCA algorithm, was constantly decreasing with read length. Click here for file

Additional file 4 **Precision and specificity of Blast based LCA is dependent on sequence length.** Using the same dataset as in Additional file 3, we measured precision (a,c) and specificity (b,d) of the taxonomic assignments. These are increasing to 100% on all taxonomic levels at higher read length, when using the full greengenes database with the exclusion of the queried sequence (a,b). When simulating that sequence related to the test sequence (≥97% identity) are absent from the database (c,d), the importance of long reads becomes more apparent. Species data is not shown in (c), as species level was not assigned and therefore the specificity was 100% in (d). *TP* true positive, *FP* false positive, *TN* true negative. Click here for file

Additional file 5: Table S1 Comparison of compositional similarity. Click here for file

Additional file 6: Table S2 Comparison of compositional and phylogenetic similarity. Click here for file

Additional file 7 **Taxa stability across pipelines.** a) The 454 datasets was analyzed in respect to taxonomic stability across the 5 pipeline versions. a) The percentage of OTU’s from different pipeline versions, which could not be assigned the respective taxonomic level. As expected, this is increasing towards the more specific levels. b) The fraction of Taxa that are present in only 1,2,3,4 or all 5 of the pipelines. Click here for file
