# Correction: LotuS: an efficient and user-friendly OTU processing pipeline

**DOI:** 10.1186/2049-2618-2-37

**Published:** 2014-10-14

**Authors:** Falk Hildebrand, Raul Y Tito, Anita Yvonne Voigt, Peer Bork, Jeroen Raes

**Affiliations:** 1 Department of Structural Biology, Vlaams Instituut voor Biotechnologie (VIB), Pleinlaan 2, Brussels 1050, Belgium; 2 Department of Bioscience Engineering, Vrije Universiteit Brussel, Pleinlaan 2, Brussels 1050, Belgium; 3 Structural & Computational Biology Unit, European Molecular Biology Laboratory (EMBL), Meyerhofstrasse 1, Heidelberg 69117, Germany; 4 Molecular Medicine Partnership Unit (MMPU), University of Heidelberg and European Molecular Biology Laboratory, Heidelberg, Germany; 5 Max Delbrück Centre for Molecular Medicine, Robert-Rössle-Str. 10, Berlin 13125, Germany; 6 Department of Microbiology and Immunology, REGA institute, KU Leuven, Herestraat 49, Leuven 3000, Belgium; 7 VIB Center for the Biology of Disease, Herestraat 49, Leuven 3000, Belgium

## Correction

The name of the second author was incorrect in the original published article. The correct author name is Raul Y Tito.
