# Supplementary material for: LotuS: an efficient and user-friendly OTU processing pipeline
Source: Microbiome. 2014 Sep 30;2:30. doi: 10.1186/2049-2618-2-30 (PMC4179863; doi:10.1186/2049-2618-2-30)
Supplement: Additional file 1 — Supplementary methods. Supplementary methods including the following sections: sample collection and 16S rRNA sequencing, 16S read simulation, comparison of pipelines and commands used to run mothur and QIIME on sample datasets. [file 2049-2618-2-30-S1.docx]

## Supplementary methods

### Sample collection & 16S rRNA sequencing

Stool samples were collected from four healthy individuals living in Germany and informed consent was obtained through the my.microbes project (<http://my.microbes.eu>). Fecal samples were collected and immediately homogenized with a clean spoon, aliquoted and stored at -80°C until DNA isolation. The DNA was isolated using ten different DNA isolation protocols. From each individual duplicates were isolated with the same protocol. The DNA was amplified according the manufacturer’s instructions targeting the V4 region of the 16S rRNA gene using primers from the NEXTflex^TM^ 16S V4 Amplicon-Seq Kit (Bioo Scientific, Austin, Texas, USA) with minor modification. For the PCR, instead of the kit’s internal polymerase the Q5 high-fidelity polymerase (New England BioLabs, Ipswich, USA) was used according to the manufacturer’s recommendations and the DNA was amplified with an annealing temperature of 56°C for 35 cycles. PCR products were cleaned up and multiplexed according to the NEXTflex^TM^ 16S V4 Amplicon-Seq Kit protocol. Sequencing was performed using a 250 bp paired-end sequencing protocol on the Illumina MiSeq platform (Illumina, San Diego, USA) at the Genomics Core Facility, European Molecular Biology Laboratory, Heidelberg. This dataset is made available online at http://psbweb05.psb.ugent.be/lotus/.

### 16S read Simulation

16S reads for length dependent taxonomic classification were simulated from 1000 greengenes database (McDonald et al., 2012). 16S genes were chosen at random, with the restriction that each species was only represented once in this 16S set and that the 16S sequence was classified to species level. Into these sequences a sequencing error was simulated, equally distributed across the read by giving each base a random chance of 0.001 to change the state to any other DNA base, equivalent to an average quality of 30. The selected sequences were removed from the greengenes database that was used in subsequent taxonomic assignments. Length restriction started from the 5’ end of sequences and subsequently more bases were added to the reference sequence to increase the simulated read length. Precision was computed = TP/(FP+TP) and Specificity = TN/(FP+TN); TN are the true negatives, that is a taxonomic level classified as unknown by the LCA, TP are taxonomic levels that were correctly identified and FP are taxonomic levels that were wrongly assigned by the LCA algorithm.

### Comparison of pipelines

We executed both mothur and QIIME without denoising sff flowgrams (the native output of a 454 sequencer), as we wanted all pipeline to start from the samefasta and quality files. However, we used a cluster based denoising in mothur, that is not dependent on flowgrams, while for QIIME to our knowledge only flowgram denoising is supported. Furthermore, in our experience sff denoising is a very time consuming step (QIIME denoising the here used experiment took several days with > 100 Cores) and we felt it would be an unfair comparison of the pipelines execution time to add this step to mothur and QIIME.

The output of the pipelines was compared with R scripts that load the data matrix and compared the richness using the R-package vegan (Oksanen et al., 2012). Bray-Curtis as well as unifrac distances were calculated from rarefied OTU abundance matrices with vegan or the function “GUniFrac” of the R-package *GUniFrac* (Chen, 2012), respectively. For unifrac distances the phylogenetic tree given by the three pipelines was used.

miSeq data richness was estimated on samples rarefied to 15,000 reads per sample, 5 paired samples had < 15,000 total reads and were excluded for all comparisons. Distances were calculated on the matrices rarefied to 15,000 reads. vegan (Oksanen et al., 2012) was used to calculate Bray-Curtis and Canberra distances; Jensen-Shannon distance was calculated from a custom implementation (Hildebrand et al., 2013). We tested the vector of 37 between sample distances between LotuS to either QR or QD using a paired t-test as implemented in R. mothur was executed on a server due to required 0.5 TB of temporary space that was not available on a Laptop. It required > 18 hours with 14 cores, until it exited with an unknown error in the make.shared step.

For reference, we include the commands used to execute QIIME and mothur on the considerably smaller 454 dataset. We followed the official QIIME tutorial on processing 454 data (http://qiime.org/tutorials/tutorial.html). Likewise, the mothur commands followed the mothur SOP (http://www.mothur.org/wiki/454_SOP).

### QIIME commands

##############################################################################

split_libraries.py -f /data/Anh.1.fna -q /data/Anh.1.qual -m /data/Map_Anh1.txt -b 10 -o /data/Map_Anh1/ -s 25 -l 200 -L 200

split_libraries.py -f /data/Anh.2.fna -q /data/Anh.2.qual -m /data/Map_Anh2.txt -b 10 -o /data/Map_Anh2/ -s 25 -l 200 -L 200 -n 1000000

cat /data/Map_Anh1/seq.fna /data/Map_Anh2/seq.fna > /data/seqtotal.fna

###close_reference:

adjust_seq_orientation.py -i /data/seqsAnh.fna -o /data/seqsAnh-Rev.fna

pick_closed_reference_otus.py -i /data/seqsAnh-Rev.fna -o /data/seqsAnh-open-ref-Rev/ -r /Users/Tomas/gg_13_5/gg_13_5_otus/rep_set/97_otus.fasta -t /Users/Tomas/gg_13_5/gg_13_5_taxonomy.txt

###open_reference:

pick_de_novo_otus.py -i /data/seqsAnh.fna -o /data/seqsAnh-short-de-novo/

##############################################################################

### mothur commands

##############################################################################

trim.seqs(fasta=/data/Anh.1.fasta, oligos=/data/Anh_mothur/map01.oligos, qfile=/data/Anh.1.qual, maxambig=0, maxhomop=8, flip=T, bdiffs=0, pdiffs=0, qwindowaverage=27, qwindowsize=50, processors=1)

trim.seqs(fasta=/data/Anh.2.fasta, oligos=/data/Anh_mothur/map02.oligos, qfile=/data/Anh.2.qual, maxambig=0, maxhomop=8, flip=T, bdiffs=0, pdiffs=0, qwindowaverage=27, qwindowsize=50, processors=1)

cat /data/Anh.1.trim.fasta /data/Anh.2.trim.fasta > /data/Anh.trim.fasta

cat /data/Anh.1.groups /data/Anh.2.groups > /data/Anh.groups

summary.seqs(fasta=Anh.trim.fasta)

unique.seqs(fasta=Anh.trim.fasta)

align.seqs(candidate=Anh.trim.unique.fasta, template=silva.bacteria.fasta, processors=1)

screen.seqs(fasta=Anh.trim.unique.align, name=Anh.trim.names, group=Anh.groups, minlength=250, end=27659, processors=1)

filter.seqs(fasta=Anh.trim.unique.good.align-silva.gold.align, vertical=T, processors=1)

chimera.slayer(fasta=Anh.trim.unique.good.filter.fasta, template=silva.gold.filter.fasta, processors=1)

remove.seqs(accnos=Anh.trim.unique.good.filter.slayer.accnos, fasta=Anh.trim.unique.good.filter.fasta, name=Anh.trim.good.names, group=Anh.good.groups)

filter.seqs(fasta=Anh.trim.unique.good.filter.pick.fasta, vertical=T, trump=., processors=1)

unique.seqs(fasta=Anh.trim.unique.good.filter.pick.filter.fasta, name=Anh.trim.good.pick.names)

pre.cluster(fasta=Anh.trim.unique.good.filter.pick.filter.unique.fasta, name=Anh.trim.unique.good.filter.pick.filter.names, diffs=1)

dist.seqs(fasta=Anh.trim.unique.good.filter.pick.filter.unique.precluster.fasta, output=lt, processors=1)

dist.seqs(fasta=Anh.trim.unique.good.filter.pick.filter.unique.precluster.fasta, cutoff=0.20, processors=1)

system(cp Anh.good.pick.groups Anh.final.groups)

system(cp Anh.trim.unique.good.filter.pick.filter.unique.precluster.phylip.dist Anh.final.dist)

system(cp Anh.trim.unique.good.filter.pick.filter.unique.precluster.names Anh.final.names)

system(cp Anh.trim.unique.good.filter.pick.filter.unique.precluster.fasta Anh.final.fasta)

cluster(method=average)

cluster(phylip=Anh.final.dist, name=Anh.final.names, cutoff=0.25, method=average)

#read.otu(list=Anh.final.an.list, group=Anh.final.groups, label=0.03)

#read.otu(shared=Anh.final.an.shared)

classify.seqs(fasta=Anh.final.fasta, name=Anh.final.names, group=Anh.final.groups, taxonomy=trainset6_032010.rdp.tax, template=trainset6_032010.rdp.fasta, cutoff=80)

get.oturep(phylip=Anh.final.phylip.dist, list=Anh.final.an.list, fasta=Anh.final.fasta)

dist.seqs(fasta=Anh.final.an.0.03.rep.fasta, output=phylip, processors=2)

clearcut(phylip=Anh.final.an.0.03.rep.phylip.dist)

##############################################################################

Bibliography

Chen, J. (2012). GUniFrac: Generalized UniFrac distances. Retrieved from http://cran.r-project.org/package=GUniFrac

Hildebrand, F., Nguyen, A. T. L., Brinkman, B., Yunta, R. G., Cauwe, B., Vandenabeele, P., … Raes, J. (2013). Inflammation-associated enterotypes, host genotype, cage and inter-individual effects drive gut microbiota variation in common laboratory mice. *Genome Biology*, *14*(1), R4. doi:10.1186/gb-2013-14-1-r4

McDonald, D., Price, M. N., Goodrich, J., Nawrocki, E. P., DeSantis, T. Z., Probst, A., … Hugenholtz, P. (2012). An improved Greengenes taxonomy with explicit ranks for ecological and evolutionary analyses of bacteria and archaea. *The ISME Journal*, *6*(3), 610–8. doi:10.1038/ismej.2011.139

Oksanen, J., Blanchet, F. G., Kindt, R., Legendre, P., Minchin, P. R., O’Hara, R. B., … Wagner, H. (2012). vegan: Community Ecology Package.
