# Supplementary figures and images for: LotuS: an efficient and user-friendly OTU processing pipeline
Source: Microbiome. 2014 Sep 30;2:30. doi: 10.1186/2049-2618-2-30 (PMC4179863; doi:10.1186/2049-2618-2-30)

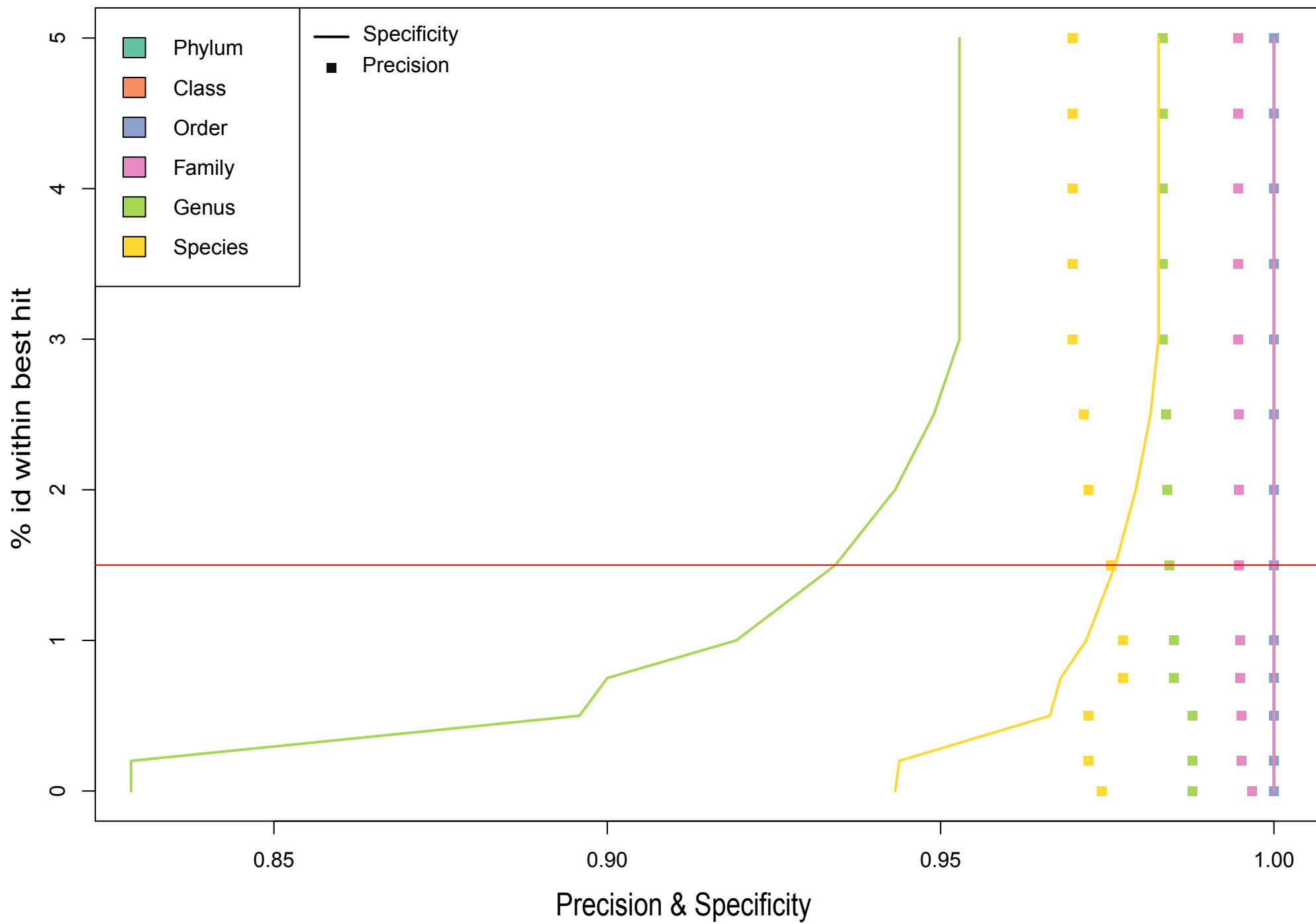

Supplement: Additional file 2 — Dependency of precision and specificity of BLAST-based LCA from best hit subset. The reference database sequences included for LCA evaluation are dependent on % identity to best hit; by default all reference sequences that have an identity ≤1.5% of the best found hit are included (red line). Specificity and precision are dependent on this parameter and the default 1.5% is a trade-off between a high precision and a high specificity. [file 2049-2618-2-30-S2.pdf]

a)

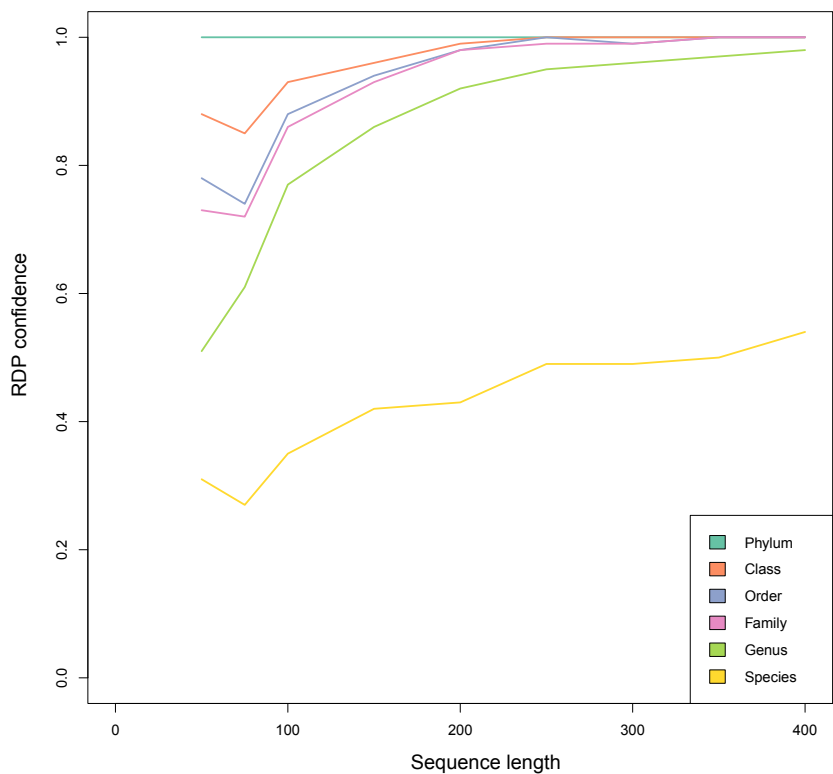

b)

Tested Sequence excluded

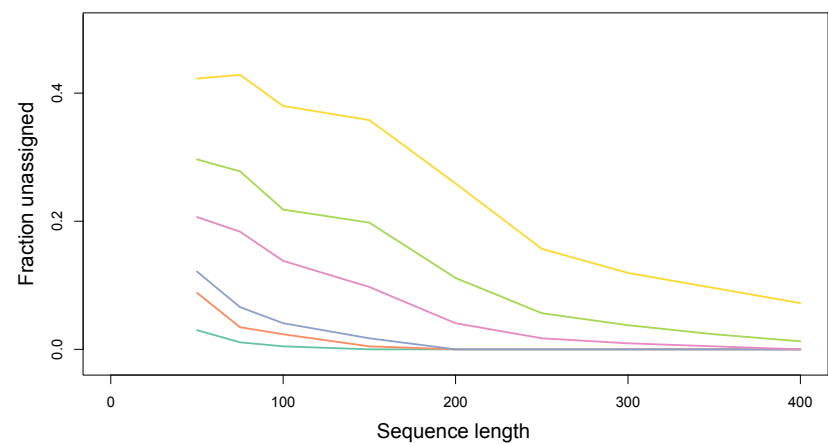

c)

Sequences 97% id to test excluded

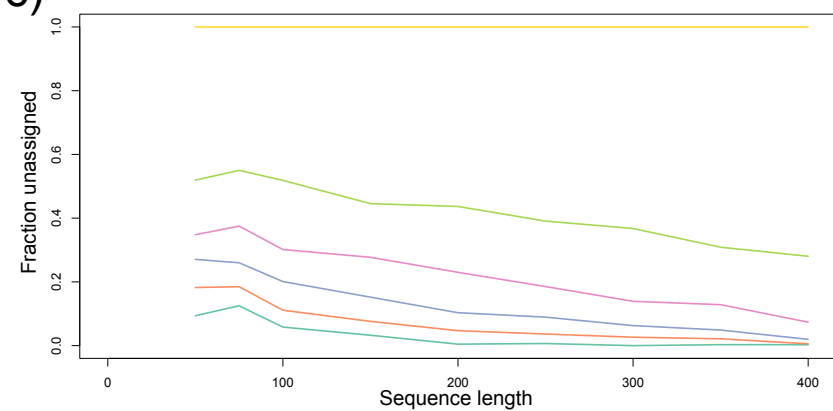

Supplement: Additional file 3 — Classification performance is dependent on 16S read length. 16S reads (1,000) of different length were simulated from the greengenes database (Additional file 1). a) RDP average classification confidence on six taxonomic levels that is increasing constantly with increasing read length. b) Similarly, the fraction of simulated reads that were not assigned to a taxon, using our LCA algorithm, was constantly decreasing with read length. [file 2049-2618-2-30-S3.pdf]
