# Supplementary material for: LotuS: an efficient and user-friendly OTU processing pipeline
Source: Microbiome. 2014 Sep 30;2:30. doi: 10.1186/2049-2618-2-30 (PMC4179863; doi:10.1186/2049-2618-2-30)
Supplement: Additional file 4 — Precision and specificity of Blast based LCA is dependent on sequence length. Using the same dataset as in Additional file 3, we measured precision (a,c) and specificity (b,d) of the taxonomic assignments. These are increasing to 100% on all taxonomic levels at higher read length, when using the full greengenes database with the exclusion of the queried sequence (a,b). When simulating that sequence related to the test sequence (≥97% identity) are absent from the database (c,d), the importance of long reads becomes more apparent. Species data is not shown in (c), as species level was not assigned and therefore the specificity was 100% in (d). TP true positive, FP false positive, TN true negative. [file 2049-2618-2-30-S4.pdf]

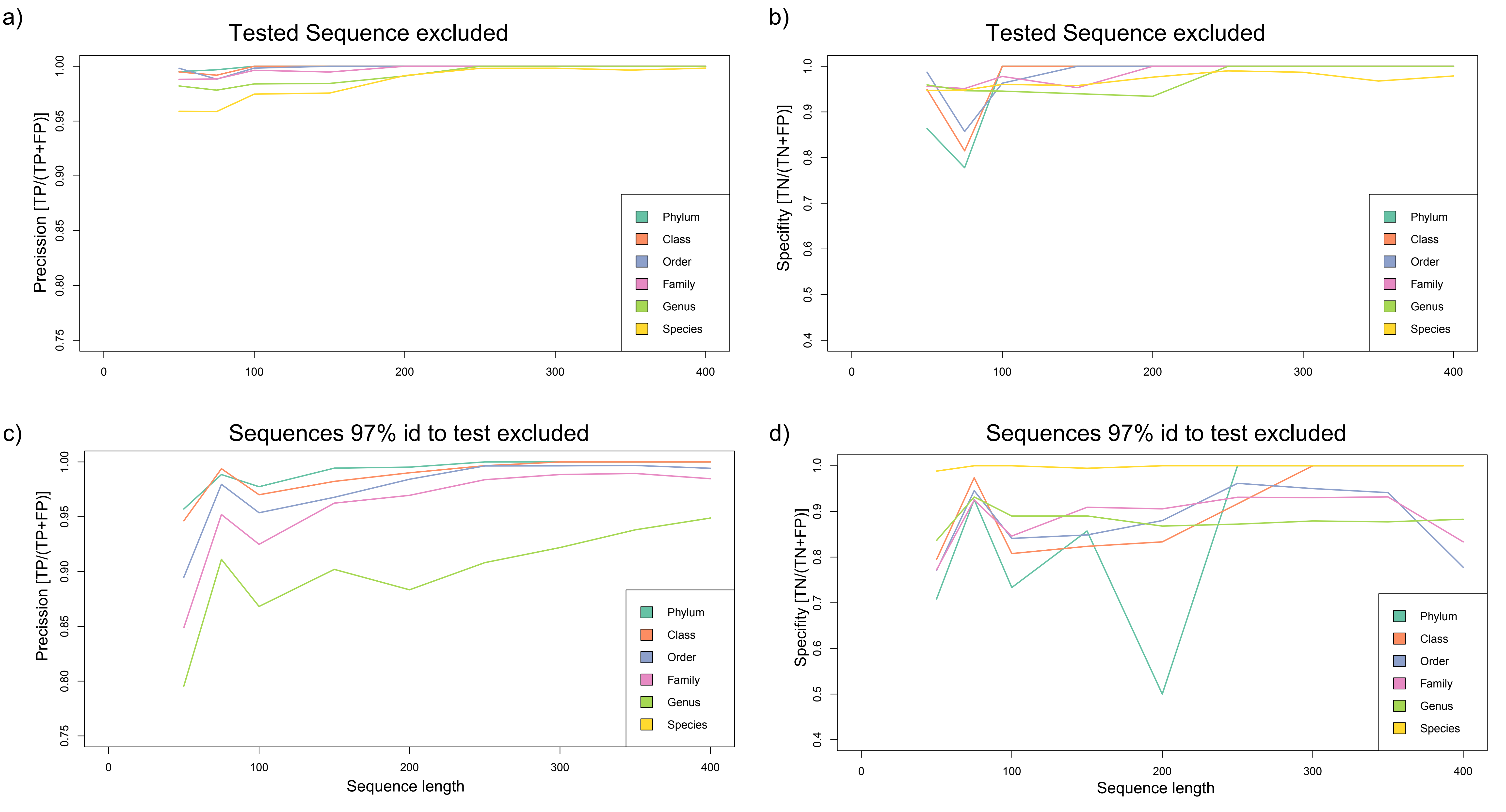

a)
Tested Sequence excluded
Precission [TP/(TP+FP)]
1.00
0.95
0.90
0.85
0.80
0.75
0
100
200
300
400
Phylum
Class
Order
Family
Genus
Species
b)
Tested Sequence excluded
Specifity [TN/(TN+FP)]
1.0
0.9
0.8
0.7
0.6
0.5
0.4
0
100
200
300
400
Phylum
Class
Order
Family
Genus
Species
c)
Sequences 97% id to test excluded
Precission [TP/(TP+FP)]
1.00
0.95
0.90
0.85
0.80
0.75
0
100
200
300
400
Sequence length
Phylum
Class
Order
Family
Genus
Species
d)
Sequences 97% id to test excluded
Specifity [TN/(TN+FP)]
1.0
0.9
0.8
0.7
0.6
0.5
0.4
0
100
200
300
400
Sequence length
Phylum
Class
Order
Family
Genus
Species
