# Supplementary material for: LotuS: an efficient and user-friendly OTU processing pipeline
Source: Microbiome. 2014 Sep 30;2:30. doi: 10.1186/2049-2618-2-30 (PMC4179863; doi:10.1186/2049-2618-2-30)
Supplement: Additional file 5: Table S1 — Comparison of compositional similarity. [file 2049-2618-2-30-S5.docx]

**Additional file 5: Table S1** Comparison of compositional similarity

|  |  |  |  |  |  |
| --- | --- | --- | --- | --- | --- |
| OTU Genus | LB | LR | QDN | QRE | MOT |
| LB | 0 | 0.9973319 | 0.9907809 | 0.9837143 | 0.9946510 |
| LR | 0. 9833921 | 0 | 0.9911778 | 0.9834094 | 0.9947204 |
| QDN | 0. 9833862 | 0.9812600 | 0 | 0.9867124 | 0.9887780 |
| QRE | 0. 9877269 | 0.9818670 | 0. 9824229 | 0 | 0.9820735 |
| MOT | 0. 9812584 | 0.9840087 | 0. 9771680 | 0.9838006 | 0 |

Average correlations between OTU and genus abundance matrices derived from three pipelines using five execution modes. The upper triangle refers to OTU level comparison, the lower triangle to genus level comparisons. *LB* LotuS BLAST, *LR* LotuS RDP, *QDN* QIIME *de novo* OTU creation, *QRE* QIIME reference OTU picking, *MOT* mothur.
