# Supplementary material for: LotuS: an efficient and user-friendly OTU processing pipeline
Source: Microbiome. 2014 Sep 30;2:30. doi: 10.1186/2049-2618-2-30 (PMC4179863; doi:10.1186/2049-2618-2-30)
Supplement: Additional file 6: Table S2 — Comparison of compositional and phylogenetic similarity. [file 2049-2618-2-30-S6.docx]

**Additional file 6: Table S2** Comparison of compositional and phylogenetic similarity

| wUF uUF | LB | LR | QDN | QRE | MOT |
| --- | --- | --- | --- | --- | --- |
| LB | 0 | 0.987237 | 0.925163 | 0.994512 | 0.959349 |
| LR | 0.96549 | 0 | 0.957658 | 0.978065 | 0.977967 |
| QDN | 0.95013 | 0.942393 | 0 | 0.917899 | 0.938077 |
| QRE | 0.957956 | 0.955813 | 0.947898 | 0 | 0.94843 |
| MOT | 0.968346 | 0.963042 | 0.953221 | 0.961168 | 0 |

Average correlations between weighted (wUF) as well as unweighted UniFrac (uUF) betadiversities between samples from two pipelines using four execution modes. The upper triangle refers to weighted UniFrac level comparison, the lower triangle to unweighted UniFrac level comparisons. *LB* LotuS BLAST, *LR* LotuS RDP, *QDN* QIIME *de novo* OTU creation, *QRE* QIIME reference OTU picking, *MOT* mothur.
